# Investigation of the Effects of Kahweol, Flurbiprofen and Co-Administration of Kahweol and Flurbiprofen on Vas Deferens, Testis and Sperm Against Ischemia-Reperfusion Injury Induced by Testicular Torsion and Detorsion in Rats

**DOI:** 10.3390/molecules31213922

**Published:** 2026-10-31

**Authors:** Okan Ali Aksoy, Serdar Moralioğlu, Yagmur Okcay, Berk Alp Goksel, Süheyla Esra Ozkocer, Kemal Tuna Olgac, Bekir Erdeve, Elif Bersu Gul, Berkay Bagci, Sema Kocabiyik, Elif Hilal Vural, Gokce Sevim Ozturk Fincan, Emre Avci, Onur Erdem, Ismail Mert Vural

**Affiliations:** 1 Department of Animal Experiments and Research Centre, Gulhane Institute of Health Sciences, University of Health Sciences, Ankara 06010, Türkiye; berkalp.goksel@sbu.edu.tr; 2 Department of Pediatric Surgery, İstanbul Zeynep Kamil Maternity and Children Diseases Health Training and Research Center, University of Health Sciences, İstanbul 34668, Türkiye; serdar.moralioglu@sbu.edu.tr; 3 Department of Pharmacology, Gulhane Faculty of Pharmacy, University of Health Sciences, Ankara 06010, Türkiye; yagmur.okcay@sbu.edu.tr (Y.O.); ismailmert.vural@sbu.edu.tr (I.M.V.); 4 Department of Medical Histology, Faculty of Medicine, Gazi University, Ankara 06500, Türkiye; sesavari@gmail.com (S.E.O.); berkay421998@gmail.com (B.B.); 5 Department of Reproduction and Artificial Insemination, Faculty of Veterinary Medicine, Ankara University, Ankara 06070, Türkiye; ktolgac@gmail.com (K.T.O.); ebersugul@gmail.com (E.B.G.); 6 Department of Pediatric Surgery, Faculty of Medicine, Hacettepe University, Ankara 06100, Türkiye; bekirerdeve@gmail.com; 7 Department of Biochemistry, Gulhane Faculty of Pharmacy, University of Health Sciences, Ankara 06010, Türkiye; semanurkayali@hotmail.com (S.K.); emre.avci@sbu.edu.tr (E.A.); 8 Department of Medical Pharmacology, Faculty of Medicine, Lokman Hekim University, Ankara 06510, Türkiye; elif.vural@lokmanhekim.edu.tr; 9 Department of Medical Pharmacology, Faculty of Medicine, Gazi University, Ankara 06500, Türkiye; gsozturk@gazi.edu.tr; 10 Deparment of Pharmaceutical Toxicology, Gulhane Faculty of Pharmacy, University of Health Sciences, Ankara 06010, Türkiye; onur.erdem@sbu.edu.tr

**Keywords:** testicular torsion, ischemia–reperfusion injury, kahweol, flurbiprofen, spermatogenesis, vas deferens

## Abstract

Unilateral testicular torsion causes ischemia–reperfusion (I/R) injury, potentially impairing testicular structure and reproductive function. This study investigated the effects of kahweol, flurbiprofen, and their combination on testicular injury and vas deferens contractility following torsion/detorsion in rats. Eighty male Wistar albino rats were divided into five groups: sham, torsion/detorsion, kahweol, flurbiprofen, and combination. Following 2 h of unilateral testicular torsion, treatments were administered immediately after detorsion and continued for six days. Histopathological, biochemical, spermatological, and isolated organ bath analyses were performed on day 7. Torsion/detorsion caused severe ipsilateral histopathological damage, increased caspase-3 immunoreactivity, and impaired vas deferens contractility. All treatments reduced caspase-3 immunoreactivity but failed to significantly improve histopathological damage. Changes in inflammatory mediators and oxidative stress markers were variable, without consistent evidence of antioxidant protection. Flurbiprofen and combination treatment partially improved noradrenaline-induced contractions, while combined treatment enhanced electrical field stimulation-induced responses in the prostatic vas deferens. Spermatological findings were limited by sample availability and variability. These findings suggest that flurbiprofen, alone or combined with kahweol, may partially improve selected functional outcomes following testicular I/R injury, although substantial structural damage persists.

## 1. Introduction

Unilateral testicular torsion is a surgical emergency caused by twisting of the spermatic cord and is typically managed by surgical detorsion. It predominantly affects newborns, children, and adolescents and may result in orchiectomy of the ipsilateral testis as well as impaired fertility of the contralateral testis [1,2]. The severity of testicular damage and associated complications depends on both the duration of torsion and the degree of testicular rotation. Torsion severe enough to induce primary ischemic degeneration is frequently followed by reperfusion injury after detorsion, a process collectively referred to as ischemia–reperfusion (I/R) injury. I/R injury is primarily mediated by the accumulation of reactive oxygen species (ROS) in the affected tissue during the ischemic phase and their subsequent oxidation upon reperfusion [3]. Inflammatory cytokine release, intracellular calcium overload, and increased thrombin activity also contribute to its pathogenesis [4]. I/R injury has been shown to impair both spermatological parameters and vas deferens motility [5]. Preventing I/R injury through adjunctive pharmacological interventions remains an active area of research. Experimental and clinical studies have demonstrated that I/R injury results in a significant deterioration of spermatological parameters [6]. Moreover, antioxidant therapies administered in conjunction with surgical intervention have been shown to mitigate the deleterious effects of I/R injury and improve spermatological outcomes [3,4,7,8].

Δ^9^-Tetrahydrocannabinol (THC), the principal psychoactive constituent of Cannabis sativa, has been used by humans for thousands of years. Cannabinoids, including THC and its derivatives, regulate a wide range of physiological processes [9]. Endogenous cannabinoids such as anandamide are lipid-based signaling molecules involved in neuromodulation, inflammation, and immune responses [10,11]. The anti-inflammatory properties of anandamide have been well documented in previous studies [12]. Kahweol, a naturally occurring diterpene found in coffee beans, has a molecular formula of C_20_H_26_O_3_, a molecular weight of 314.4 g/mol, and a polycyclic diterpene skeleton containing a furan ring, structurally resembling cafestol. The main structural difference is the presence of an additional conjugated double bond in kahweol, which may contribute to differences in their biological activities [13]. Kahweol has been proposed to influence endocannabinoid signaling through FAAH-related mechanisms and has been reported to exert anti-inflammatory, hepatoprotective, anticancer, and antidiabetic effects [13,14] and to attenuate neuroinflammation [15]. The pharmacokinetic properties of kahweol have also recently been investigated by our group in an in vivo rabbit model [16].

Flurbiprofen, a nonsteroidal anti-inflammatory drug (NSAID) belonging to the arylpropionic acid derivatives, has a molecular formula of C_15_H_13_FO_2_, a molecular weight of 244.3 g/mol, and a biphenyl structure containing a fluorine substituent and a propionic acid moiety. It exerts its anti-inflammatory and analgesic effects primarily through the inhibition of cyclooxygenase (COX) enzymes, thereby reducing prostaglandin synthesis [17,18,19]. A previous study demonstrated a complex interaction between endocannabinoids and prostaglandins in the regulation of vas deferens contractility, showing that flurbiprofen potentiates anandamide-induced contractile responses while reducing FAAH levels [20]. The primary research question was whether kahweol, flurbiprofen, or their combination attenuates testicular injury and preserves vas deferens contractility seven days after testicular torsion/detorsion in rats. Histological, apoptotic, biochemical, and spermatological outcomes were assessed, with cyclooxygenase- and endocannabinoid-related markers examined on an exploratory basis.

## 2. Materials and Methods

### 2.1. Animals and Groups

The male Wistar Albino rats used in this study were bred, raised, and maintained at the Department of Animal Experiments and Research Centre, Gülhane Institute of Health Sciences, University of Health Sciences, Ankara, Türkiye. All experimental procedures were conducted at the same facility. The study was approved by the Gülhane Animal Experiments Local Ethics Committee of the University of Health Sciences, Türkiye (Gülhane HADYEK; approval number: ETIK-2024/17; approval date: 15 August 2024). The animals were housed in standard laboratory cages under controlled environmental conditions, including a temperature of 21–24 °C, approximately 50% relative humidity, and a 12 h automated light/dark cycle. Standard laboratory feed and drinking water were provided ad libitum throughout the experimental period.

The experimental animals were 12-week-old male Wistar Albino rats weighing 250–300 g. At least one week before the initiation of the experimental procedures, a total of 80 rats were allocated to five experimental groups (*n* = 16 per group): Sham, Torsion/Detorsion (I/R), Kahweol, Flurbiprofen, and Kahweol + Flurbiprofen. Group allocation was performed through informal, arbitrary selection without the use of a computer-generated randomization sequence, random number table, or other formal random allocation procedure. Thus, no formal randomization method was employed. Detailed allocation information is given in Table S1. All animals were allowed at least one week to acclimatize to the experimental environment before the procedures began.

Sample size estimation using OpenEpi Version 3 for a two-means comparison, based on assumed means and standard deviations, indicated six animals per group at 95% confidence and 80% power. Allowing for approximately 20% anticipated perioperative or postoperative attrition, eight animals were allocated to each analytical subgroup. Animals in each group were further divided into two subgroups (*n* = 8 each): one for isolated organ bath and histological analyses, and the other for spermatological and biochemical analyses. Vas deferens tissues were used for organ bath experiments, with corresponding testes evaluated histologically. Blood and tissue samples from the remaining animals were used for ELISA, biochemical, and sperm analyses. This study was conducted in accordance with the ARRIVE 2.0 guidelines.

Rats were anesthetized with intraperitoneal ketamine (80 mg/kg) and xylazine (10 mg/kg), with doses calculated individually according to each animal’s body weight. The injection was administered into the right lower abdominal quadrant at a 30–45° angle after negative aspiration. Anesthetic depth was confirmed by the absence of responses to skin and toe pinch stimuli. The surgical site was prepared with povidone–iodine, and surgery was performed under aseptic conditions. A 1 cm right scrotal incision was made, and the right testis was exposed while preserving the spermatic cord and vessels. Testicular torsion was induced by rotating the testis 720° clockwise and fixing it to the scrotal wall with 3/0 non-absorbable sutures. After 2 h of ischemia, detorsion was performed. The torsion group received vehicle (10% dimethyl sulfoxide [DMSO] in physiological saline), whereas treatment groups received intrascrotal kahweol (5 mg/kg), flurbiprofen (10 mg/kg), or their combination immediately after detorsion. A 10% DMSO solution was used as the vehicle in all experimental groups because of the solubility requirements of the compounds. In the sham group, the testis was exposed, rotated 720° clockwise, immediately detorsioned, and the vehicle was administered intrascrotally using a 26G insulin syringe. For six days after surgery, sham and torsion groups received vehicle (10% DMSO), whereas treatment groups received daily intraperitoneal injections of kahweol (1 mg/kg), flurbiprofen (10 mg/kg), or their combination. Doses were selected based on previous studies [15,18]. Kahweol and flurbiprofen were administered at an injection volume of 1 mL/kg for both routes, with doses and injection volumes calculated individually according to each animal’s body weight. The same injection volume was used for vehicle administration. At a DMSO concentration of 10% (*v*/*v*), each administration delivered 0.1 mL/kg DMSO, corresponding to a cumulative nominal volume of 0.7 mL/kg over seven administrations (one intrascrotal and six intraperitoneal).

#### 2.1.1. Blinding Procedures

The investigators performing biochemical, histological, spermatological, and isolated organ bath analyses were blinded to the experimental group assignments during outcome assessment. Group allocation information was not disclosed to these investigators until the analytical procedures had been completed and the results had been obtained. In contrast, the investigators responsible for surgical procedures, postoperative animal care, drug preparation and administration, and euthanasia were aware of the group assignments. Therefore, blinding was implemented at the outcome assessment stage rather than throughout the entire experimental procedure.

#### 2.1.2. Humane Endpoints

The following humane endpoints were predefined in the approved animal ethics protocol: body weight loss exceeding 15%; impaired locomotion; inadequate food or water intake; markedly reduced responsiveness to external stimuli; and any condition requiring humane intervention or termination based on the attending veterinarian’s assessment. None of the animals met these criteria, and all 80 rats completed the planned experimental period without early humane termination. However, sperm samples could not be obtained from some animals because of severe testicular and epididymal damage, resulting in missing spermatological data and unequal sample sizes for these outcomes.

#### 2.1.3. Postoperative Analgesia and Veterinary Care

According to the approved animal ethics protocol, no additional standardized postoperative analgesic regimen was included in the experimental design. The designated experimental treatments were administered daily throughout the postoperative period. The protocol stipulated that any animal requiring veterinary intervention would receive prompt assessment and appropriate treatment, with early humane termination considered when necessary to prevent prolonged suffering.

#### 2.1.4. Postoperative Monitoring and Animal Welfare

Throughout the postoperative experimental period, all animals were monitored twice daily to assess their general health status, food and water intake, and overall well-being. No deterioration in general condition, food or water intake, or body weight loss was observed in any animal during the monitoring period.

#### 2.1.5. Euthanasia Procedure

Twenty-four hours after the sixth injection, all animals were placed under general anesthesia, and intracardiac blood samples were collected while they remained anesthetized. Subsequently, euthanasia was performed by cervical dislocation under general anesthesia, in accordance with the protocol approved by the Gülhane Animal Experiments Local Ethics Committee (Gülhane HADYEK; approval number: ETIK-2024/17). Following euthanasia, bilateral vas deferens and testicular tissues were collected, and sperm collection was attempted for the planned analyses. Serum was obtained by centrifugation of the collected blood samples. Tissue samples designated for biochemical analyses were rinsed with cold phosphate-buffered saline (PBS) and stored at −80 °C until analysis.

### 2.2. Histological Examination

Testicular tissues were fixed in Bouin’s solution, embedded in paraffin, sectioned at 5 μm, and stained with hematoxylin and eosin (H&E). Histopathological evaluation was performed using the modified Cosentino scoring system on 100 seminiferous tubules per sample [21,22]. Each tubule was graded on a scale from 1 to 4 based on the severity of the damage, as follows: Grade 1 indicated normal tissue with regularly organized germ cells; Grade 2 represented testicular damage with less organized germ cells; Grade 3 was characterized by desquamation of germ cells with indistinct tubule borders; Grade 4 showed severe testicular damage with extensive germ cell necrosis. The Cosentino score for each specimen was then calculated by dividing the sum of the products of each grade frequency and its corresponding score by the total number of evaluated tubules (100). For immunohistochemical analysis, antigen retrieval and endogenous peroxidase blocking were performed before incubation with anti-caspase-3 antibody (active/cleaved, 1:1000, NB100-56113, Novus Biologicals, Centennial, CO, USA). Sections were then incubated with a biotinylated secondary antibody and streptavidin–peroxidase according to the manufacturers’ instructions. For immunohistochemical quality control, negative control sections were processed using all identical steps with the omission of the primary antibody. Immunoreactivity was visualized with AEC, counterstained with hematoxylin, and quantified using ImageJ (version 1.54p) (NIH, USA). Caspase-3 expression was scored as 0 (none), 1 (weak), 2 (moderate), or 3 (strong), and the H-score was calculated using the formula: H-score = ΣPi(i + 1) [22]. Histopathological and immunohistochemical evaluations were performed in a blind manner by an experienced histologist unaware of the experimental groups.

### 2.3. Measurement of Enzymes, Mediators, and Oxidative Stress Biomarkers

#### 2.3.1. Tissue Homogenization and Biochemical Analyses

Testicular tissue samples were weighed before homogenization and processed in 1.5 mL of phosphate-buffered saline (PBS; pH 7.4) using a mechanical tissue homogenizer at 6000 rpm for 60 s. The homogenates were subsequently centrifuged at 5000 rpm for 5 min under refrigerated conditions. The resulting supernatants were collected and used for biochemical analyses. The tissue preparation procedure was based on the methodological approach described by Atasever et al. (2026) [23]. Testicular tissue samples were weighed before homogenization and processed in 1.5 mL of phosphate-buffered saline (PBS; pH 7.4) using a mechanical tissue homogenizer at 6000 rpm for 60 s. The homogenates were subsequently centrifuged at 5000 rpm for 5 min under refrigerated conditions, and the resulting supernatants were collected for biochemical analyses. The tissue preparation procedure was based on the methodological approach described by Atasever et al. (2026) [23]. The same buffer volume and processing conditions were used for all samples. However, the individual tissue-weight records were unavailable for retrospective verification. Consequently, equivalent starting tissue masses and tissue-to-buffer ratios could not be confirmed. Although the homogenization procedure was consistent, quantitative equivalence of tissue input across samples could not be established.

The concentrations of cyclooxygenase-1 (COX-1), cyclooxygenase-2 (COX-2), fatty acid amide hydrolase (FAAH), prostaglandin E2 (PGE2), anandamide (AEA), prostaglandin E1 (PGE1), tumor necrosis factor-alpha (TNF-α), interleukin-1 alpha (IL-1α), interleukin-6 (IL-6), prostaglandin F2α (PGF2α), and neopterin were determined in serum and tissue homogenate samples using commercially available ELISA kits (Bioassay Technology Laboratory [BT-LAB], Shanghai, China), according to the manufacturers’ instructions. The kit catalog numbers were E1245Ra for COX-1, E0296Ra for COX-2, E1961Ra for FAAH, E0504Ra for PGE2, EA0024Ge for AEA, EA0152Ra for PGE1, E076Ra for TNF-α, E0118Ra for IL-1α, E0135Ra for IL-6, EA0151Ra for PGF2α, and E0958Ra for neopterin. The analytical sensitivities and measurement ranges were 0.113 ng/mL and 0.2–60 ng/mL for COX-1; 0.026 ng/mL and 0.05–20 ng/mL for COX-2; 0.026 ng/mL and 0.05–20 ng/mL for FAAH; 0.026 ng/mL and 0.05–15 ng/mL for PGE2; 2.63 ng/mL and 5–320 ng/mL for AEA; 0.06 ng/mL and 0.13–8 ng/mL for PGE1; 2.51 ng/L and 5–1000 ng/L for TNF-α; 0.48 ng/L and 1–300 ng/L for IL-1α; 1.87 ng/L; 0.052 ng/L and 0.1–40 ng/L for IL-6 and 3.75–240 ng/L for PGF2α; and 0.03 nmol/L and 0.05–30 nmol/L for neopterin, respectively. According to the supplied kit specifications, the intra-assay and inter-assay coefficients of variation were below 8% and 10%, respectively, for COX-1, COX-2, FAAH, PGE2, TNF-α, IL-1α, and neopterin. The corresponding values were below 10% and 12%, respectively, for AEA, PGE1, and PGF2α. These values represent manufacturer-reported analytical precision rather than experimentally determined coefficients of variation for the present study.

For anandamide (AEA; EA0024Ge) and prostaglandin E1 (PGE1; EA0152Ra) determinations, samples were diluted 1.5-fold before analysis. The corresponding dilution factors were taken into account when calculating the final analyte concentrations. ELISA measurements were performed in duplicate, and the mean of the duplicate measurements was used for subsequent statistical analyses. The remaining assays were performed according to the corresponding manufacturers’ instructions. ELISA absorbance was measured using a Bio-Tek ELx800 microplate reader, and washing steps were performed using a Bio-Tek ELx50 automated strip washer. Analyte concentrations were calculated using the corresponding calibration curves.

The immunoassay configurations were considered individually according to the available kit-specific documentation. Anandamide (AEA; EA0024Ge) was measured using a competitive ELISA, as described in the supplier’s product information. Prostaglandin E2 (PGE2; E0504Ra) was assessed using a manufacturer-designated sandwich ELISA. The available PGE1 protocol also described a sandwich-type antibody-capture procedure. The assay configurations of other small-molecule analytes were not assumed in the absence of definitive kit-specific documentation. Because small-molecule immunoassays may be subject to analytical specificity and cross-reactivity limitations, the measured AEA and prostaglandin concentrations were interpreted as immunoassay-derived analyte levels.

Total antioxidant status (TAS; RL0017), total oxidant status (TOS; RL0024), and paraoxonase-1 (PON-1; RL0031) were determined using commercial colorimetric assay kits (Rel Assay). Malondialdehyde (MDA; Otto1001), superoxide dismutase (SOD; Otto3047), myeloperoxidase (MPO; Otto3048) glutathione peroxidase (GPx; Otto2085), and glutathione (GSH; Otto3053) were assessed using commercial colorimetric assay kits (Otto Scientific). TAS, TOS, PON-1, SOD, MPO, GPx, and GSH were analyzed using a Mindray BS400 automated analyzer, whereas MDA was measured using a Rel Assay microplate reader. GSH was measured in serum samples, while the other colorimetric parameters were assessed in serum and tissue samples. TAS and TOS were measured using endpoint colorimetric methods at 660 and 546 nm, respectively. Their analytical measurement ranges were 0.1–3.5 mmol/L for TAS and 0.2–80 µmol/L for TOS. The intra-assay and inter-assay coefficients of variation were 2.8% and 3.3% for TAS, respectively, and 3.2% and 3.9% for TOS, respectively. ELISA-based measurements of COX-1, COX-2, and FAAH reflect immunoreactive protein concentrations rather than direct catalytic enzyme activity. Accordingly, changes in these parameters were interpreted as changes in measured protein levels and not as direct evidence of enzymatic activation or inhibition.

#### 2.3.2. Glutathione-Related Parameters and Oxidative Stress Index

Glutathione peroxidase (GPx) activity was determined in serum and testicular tissue homogenates using a commercial colorimetric assay kit (Otto Scientific, catalog no. Otto2085) on a Mindray BS400 automated analyzer. The assay was based on the method of Paglia and Valentine, in which GPx catalyzes the oxidation of reduced glutathione by cumene hydroperoxide. Oxidized glutathione is subsequently reduced in a coupled reaction involving glutathione reductase and NADPH. The decrease in NADPH absorbance at 340 nm was monitored, and GPx activity was expressed in U/L.

The oxidative stress index (OSI) was calculated as the ratio of total oxidant status (TOS) to total antioxidant status (TAS). TOS was expressed in µmol H_2_O_2_ equivalent/L, whereas TAS was expressed in mmol Trolox equivalent/L. Before calculating OSI, TAS values were converted from mmol/L to µmol/L. OSI (arbitrary units) = TOS (µmol H_2_O_2_ equivalent/L)/TAS (µmol Trolox equivalent/L). Accordingly, OSI was reported in arbitrary units (AU).

#### 2.3.3. Reduced Glutathione (GSH) and Oxidized Glutathione (GSSG) Measurements

GSH and GSSG concentrations were determined in serum samples using a colorimetric method based on the modified Ellman procedure. GSH was measured using a commercial assay kit (Otto Scientific, catalog no. Otto3053) on the Mindray BS400 automated biochemistry analyzer. The assay involved the reaction of glutathione thiol groups with 5,5′-dithiobis(2-nitrobenzoic acid) (DTNB), producing a colored compound measured spectrophotometrically at 412 nm.

For total glutathione determination, oxidized glutathione was reduced to GSH using sodium borohydride (NaBH_4_) under alkaline conditions. Hydrochloric acid was subsequently added to prevent interference from residual reducing agent during the DTNB reaction. GSSG concentrations were calculated by subtracting reduced glutathione concentrations from total glutathione concentrations and dividing the difference by two. Total glutathione was expressed as GSH equivalents. The following equation was used:

GSSG (mmol/L) = [Total glutathione (mmol/L) − GSH (mmol/L)]/2

Appropriate dilution factors were taken into account in the calculations. Both GSH and GSSG concentrations were expressed in mmol/L.

#### 2.3.4. Expression and Normalization of Tissue Biochemical Results

Biochemical measurements in testicular tissue homogenates were performed using the supernatants obtained after centrifugation. The final assay-derived concentrations and enzyme activities were used for statistical analysis and reported in their respective assay-specific units (e.g., ng/L, nmol/L, mmol/L, µmol/L, U/L, or U/mL). No additional normalization to tissue wet weight or total protein content was performed.

Because the individual tissue-weight records were unavailable, the amount of tissue represented in each homogenate could not be verified retrospectively. Moreover, all available supernatants had been consumed during the planned biochemical assays, leaving no residual material for additional protein determination. Thus, retrospective normalization was not possible, and quantitative comparability of the tissue biochemical measurements across experimental groups and tissue sides could not be confirmed.

### 2.4. Spermatological Analyses

Epididymal Sperm Extraction: Following the sacrifice of the animals, the cauda epididymides belonging to the affected and unaffected testes were carefully isolated. The epididymis samples were cleared of blood and other tissue residues and immediately transferred into 3 mL of sperm analysis solution (3.025 g TRIS, 1.7 g Citric Acid, 1.25 g Glucose, 150,000 IU Penicillin G, 150 mg Streptomycin, 100 mL ultra-distilled water) at 37 °C. The cauda epididymis tissue was incised at multiple points using a sharp scalpel, while immersed in the sperm analysis solution, and directly incubated at 38.5 °C for 15 min to allow the spermatozoa to migrate into the solution [3].

Sperm Motility: Motility and kinematic characteristics in the semen samples were analyzed by a Computer-Assisted Sperm Analysis (SCA^®^ v.4.2, Barcelona, Spain) system [3]. At the end of the incubation period, 5 μL of the sperm sample was placed on a slide, covered with a 22 × 22 mm coverslip, and analyzed using a phase-contrast microscope (Nikon ECLIPSE 50i, Tokyo, Japan) equipped with a heating stage (37 °C) and a camera (Basler^®^). Images were captured at 10× magnification from 5 different fields for each sample. The analysis evaluated total motility (TMOT; %), progressive motility (PMOT; %), Velcity curved line (VCL; μm/s), Velocity straight line (VSL; μm/s), Velocity average path (VAP; μm/s), Linearity (LIN; %), Straightness (STR; %), Wobbling (WOB; %), Beat cross frequency (BCF, Hz), Amplitude of lateral head displacement (ALH, μm) and hyperactivity (%) parameters. Particle dimension range was arranged between 200 and 1600 μm2. Average of 100–200 spermatozoa were analyzed in each microscopic field. Spermatozoa with the straightness higher than 70% were considered as progressive [24].

Determination of Sperm Concentration: The sperm concentration was determined by the hemocytometric method. For this procedure, 10 µL was taken from the sperm samples obtained after incubation and fixed with a fixation solution (0.25 g sodium bicarbonate, 0.5 mL 37% formalin, 50 mL ultra-distilled water) at a ratio of 1:20 (v:v) [3]. From the fixed sperm samples, 10 µL was loaded into the upper and lower chambers of a Thoma counting slide, and the slide was left to settle for 5–10 min. Subsequently, spermatozoa within the boundaries of 10 large squares (5 from the lower section and 5 from the upper section) were counted, and the sperm concentration (×106 per mL) was calculated according to Tırpan (2022) [25].

Abnormal Sperm Rate: The Diff-Quik rapid staining method was applied to determine the rate of abnormal spermatozoa. In this method, a smear was prepared on a slide using 10 µL of the sperm sample obtained after incubation. The prepared sperm smears were air-dried for a few seconds, then sequentially dipped into Diff-Quik (DiffPlus Fast, Alcom, İstanbul, Türkiye) reagents 1, 2, and 3 for 10 s each, rinsed with distilled water to remove excess stain, and left to dry. The dried preparations were examined under a microscope at 200× magnification. The spermatozoa were evaluated as normal or abnormal based on the morphological status of the head, midpiece, tail structures and cytoplasmic droplets. A minimum of 200 spermatozoa were counted, and morphologically normal and abnormal spermatozoa (%) were determined [4].

Dead Sperm Rate (Viability): The eosin staining method was utilized to determine the dead spermatozoa rate [4]. For this method, 5 µL of the sperm sample obtained after incubation was placed on a slide warmed to 37 °C, and an equal volume of eosin stain was added to the drop. The mixture was kept on a heating stage at 37 °C for a few seconds, then a smear was prepared from the sperm–stain mixture and left to dry on the heating stage at 37 °C. The dried preparations were examined under a microscope at 200× magnification. Spermatozoa with red/dark pink heads were evaluated as dead, while those with colorless/light pink heads were evaluated as live. At least 200 spermatozoa were counted, and dead and live spermatozoa rates (%) were determined [4].

### 2.5. Isolated Organ Bath Experiments

After sacrifice, a midline abdominal incision was made, and bilateral vas deferens tissues were dissected at the prostatic and epididymal ends near the inguinal canal, immediately placed in Krebs–Henseleit solution, and transferred to the laboratory. Each vas deferens was divided into equal prostatic and epididymal segments and prepared as two strips. The strips were mounted in 10 mL organ baths containing Krebs–Henseleit solution, continuously gassed with 95% O_2_ and 5% CO_2_, and maintained at 37 °C (pH 7.35–7.45). Tissues were connected to a force transducer (FDT10-A, May IOBS 99; COMMAT Communication Co., Ltd.), and isometric contractions were recorded using a multi-channel acquisition system (MP36B-CE) and BSL PRO software (v3.6.7; BIOPAC Systems Inc., Santa Barbara, CA, USA). Preparations were set to a resting tension of 1 g and allowed to equilibrate for 60 min, with bath solution changes every 15 min. Following equilibration, tissue viability and contractile responsiveness were assessed by administration of 80 mM KCl. The preparations were subsequently washed every 10 min and allowed to recover for 30 min until the resting tension returned to baseline. Electrical field stimulation (EFS) was applied through platinum electrodes to evaluate electrically evoked contractile responses. Stimulation was performed at frequencies of 2, 4, 8, 16, and 32 Hz, using a voltage of 60 V, a pulse duration of 1 ms, and a stimulation period of 10 s. A single stimulation train was applied at each frequency, with an interval of 6 min between successive stimulations. Isometric contractile responses were recorded at each stimulation frequency. Following completion of the EFS protocol, the preparations were washed with fresh Krebs–Henseleit solution every 15 min and allowed to rest for 60 min. Cumulative concentration–response curves to noradrenaline (10^−7^–3 × 10^−4^ M) were then obtained. Each subsequent concentration was added after the contractile response to the preceding concentration had stabilized. After completion of the noradrenaline concentration–response experiments, the preparations were washed and allowed an additional 60 min recovery period. Subsequently, cumulative concentration–response curves to α,β-methylene ATP (10^−7^–10^−3^ M) were obtained. Contractile responses were recorded separately for the ipsilateral and contralateral prostatic and epididymal segments to evaluate the effects of testicular ischemia–reperfusion injury and the administered treatments on vas deferens contractility.

### 2.6. Drugs and Solutions

Flurbiprofen, a nonselective COX inhibitor, was used as the racemic (±) mixture (purity ≥ 99%). Flurbiprofen and kahweol were purchased from Cayman Chemical Company (Ann Arbor, MI, USA), whereas noradrenaline and α,β-methylene ATP were purchased from Sigma (St. Louis, MO, USA). The Krebs–Henseleit solution was prepared daily based on previous studies [20]. The composition of the Krebs–Henseleit solution was (mM) NaCl 118; KCl 4.7; NaHCO_3_ 25; NaH_2_PO_4_-2H_2_O 0.9; CaCl_2_-2H_2_O 1.26; MgCl-6H_2_O 0.5; and glucose monohydrate 11.

### 2.7. Statistical Analysis

Statistical analyses were performed using GraphPad Prism (ver. 10) software. Cosentino scores and H-score were analysed separately using one-way ANOVA and Tukey’s multiple comparisons without pairing the ipsilateral and the contralateral testes. Data are presented as mean ± SEM, unless specified otherwise. Normality was assessed using the Shapiro–Wilk test. Independent groups were compared using one-way ANOVA followed by Tukey’s test or the Kruskal–Wallis test followed by Dunn’s test, as appropriate. Paired ipsilateral and contralateral measurements were analyzed using two-way repeated-measures ANOVA; a mixed-effects model was used when paired values were missing. Multiplicity-adjusted *p* values were reported for post hoc comparisons. Missing values were not imputed, and *p* < 0.05 was considered statistically significant. Detailed quantitative results and corresponding sample sizes are provided in the main and Supplementary Materials.

## 3. Results

### 3.1. Histological Examination

**Figure 1:**
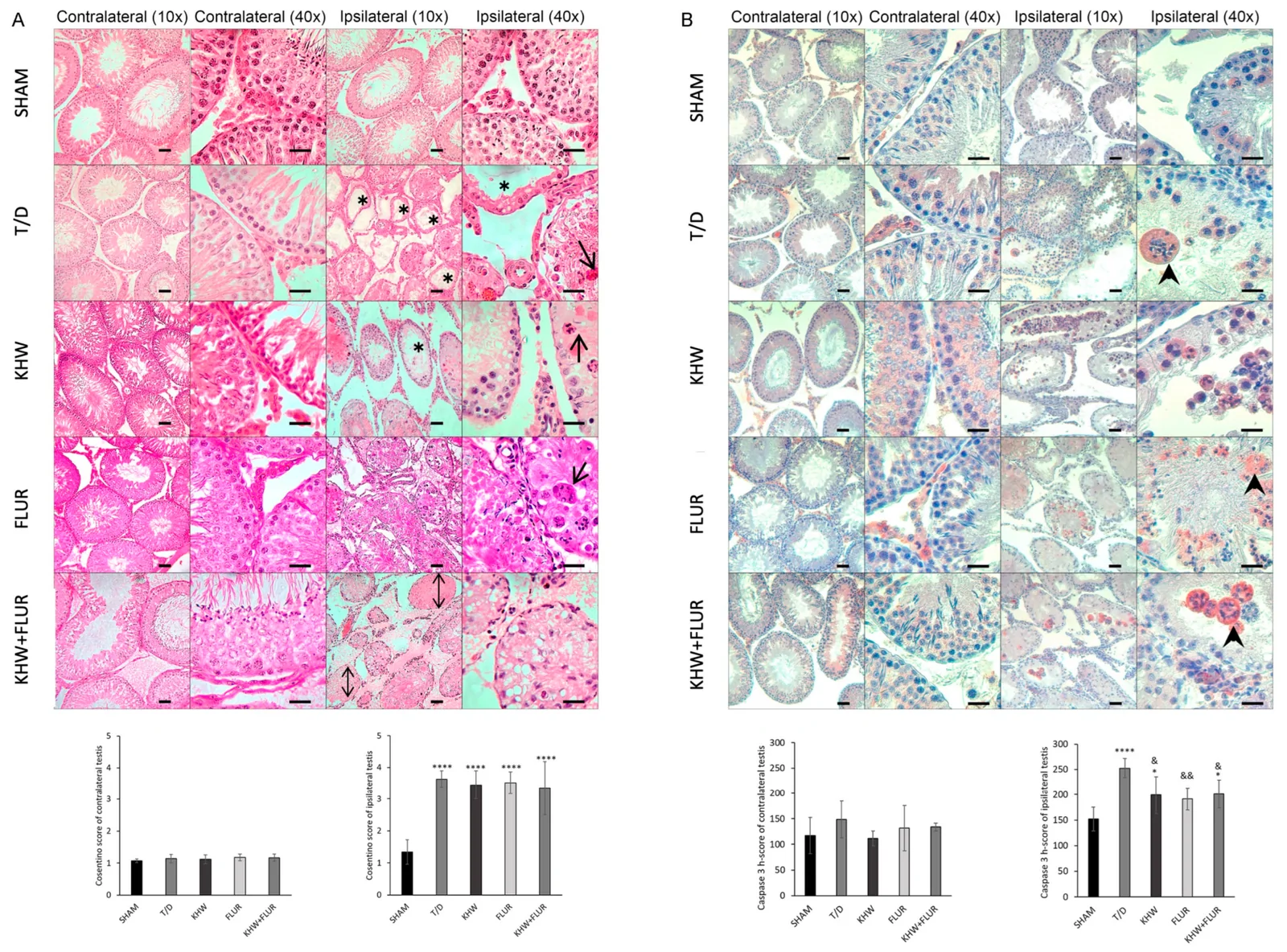
Histopathological and immunohistochemical evaluation of testicular tissues following experimental testicular torsion/detorsion. (**A**) Representative hematoxylin and eosin (H&E)-stained micrographs and corresponding Cosentino scores of contralateral and ipsilateral testes. Scale bars are located at the right bottom of each micrograph, which represent 50 µm for images obtained with a 10× objective and 20 µm for images obtained with a 40× objective. The lumens of the seminiferous tubules showing a decrease in wall thickness due to germinal epithelium desquamation are indicated by asterisks (*). Cells with dark eosinophilic cytoplasm and multiple nuclei, resulting from germinal epithelium dissociation, are indicated by arrows. Seminiferous tubules exhibiting eosinophilic cytoplasm and cells with lost nuclear integrity are indicated by double-headed arrows. Data are presented as bar plots (mean ± SD). No significant differences were observed among the groups in the contralateral testes, whereas the ipsilateral testes of the sham group showed a significantly lower Cosentino score compared with the other groups (*p* < 0.0001) (**B**) Representative immunohistochemical staining for Caspase-3 and corresponding quantitative analysis. Scale bars are located at the right bottom of each micrograph, which represent 50 µm for images obtained with a 10× objective and 20 µm for images obtained with a 40× objective. Arrowheads (>) indicate desquamated cells with multiple nuclei showing positive immune reaction with anti-caspase 3 antibody. Data are presented as bar plots (mean ± SD). * *p* < 0.05, **** *p* < 0.0001 significant difference compared to the sham group; ^&^ *p* < 0.05, ^&&^ *p* < 0.01 significant difference compared to the T/D group. SHAM (*n* = 6), T/D: Torsion/detorsion (*n* = 6), KHW: Kahweol (*n* = 6), FLUR: Flurbiprofen (*n* = 6), KHW + FLUR: Kahweol+ Flurbiprofen (*n* = 7).

The structural integrity of the contralateral testis was preserved in all groups, with regular seminiferous tubules with intact germinal epithelium. In contrast, ischemia/reperfusion (I/R) injury induced marked morphological alterations in the ipsilateral testis. These included desquamation of germ cells into tubular lumen, desquamated cells with eosinophilic cytoplasm with multiple nuclei, and seminiferous tubules filled with the anucleate eosinophilic mass, consistent with the remnants of necrotic germinal epithelium (Figure 1). These findings resulted in a significantly higher Cosentino score in the ipsilateral testes of the T/D group compared with the sham group (*p* < 0.0001). None of the applied treatments restored the morphological findings to normal, and none was effective in reducing the Cosentino scores. KHW, FLUR, and KHW + FLUR groups all had significantly higher Cosentino scores than the sham group (*p* < 0.0001), whereas no significant difference was found between the T/D group and any of the treatment groups (*p* > 0.05).

Caspase-3 immunoreactivity was significantly increased after torsion/detorsion and was reduced by kahweol, flurbiprofen, and their combination. No significant differences in caspase-3 expression were observed among contralateral testes. (Figure 1). Detailed comparisons among the experimental groups are provided in the Supplementary Material.

### 3.2. Measurement of Enzymes, Mediators, and Oxidative Stress Biomarkers

**Figure 2:**
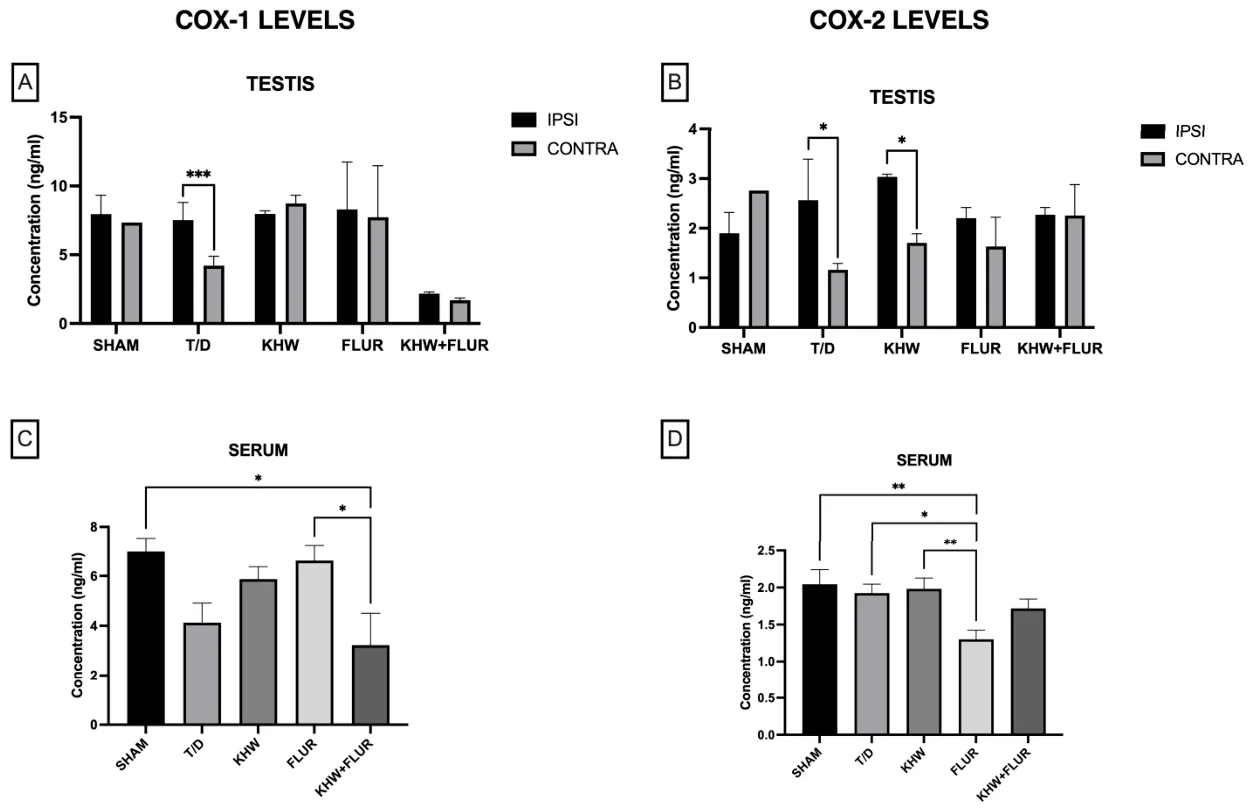
COX-1 and COX-2 levels in testis and serum. (**A**) COX-1 levels in testis, (**B**) COX-2 levels in testis, (**C**) COX-1 levels in serum, (**D**) COX-2 levels in serum. Data are presented as mean ± S.E.M. Black bars represent ipsilateral (IPSI) tissues, whereas gray bars represent contralateral (CONTRA) tissues. Testicular data were analyzed using mixed-effects ANOVA followed by Tukey’s post hoc test, whereas serum data were analyzed using one-way ANOVA followed by Tukey’s post hoc test. * *p* < 0.05, ** *p* < 0.01, and *** *p* < 0.001 indicate statistically significant differences (*n* = 4 per group for testicular tissues and *n* = 5–6 per group for blood samples). Statistical comparisons were performed among all experimental groups in each panel. Only statistically significant comparisons are indicated; comparisons without significance indicators were not statistically significant (*p* ≥ 0.05).

Testicular COX-1 and COX-2 levels showed ipsilateral increases following torsion/detorsion, whereas these differences were attenuated by treatment (*p* < 0.05). In serum, flurbiprofen significantly reduced COX-2 levels, while the combined treatment decreased COX-1 levels. (Figure 2).

No significant changes were observed in testicular FAAH or anandamide levels. In serum, FAAH levels were significantly increased in the kahweol group, whereas anandamide remained unchanged (Figure S1). No significant differences were detected in PGE1, PGF2α, or serum PGE2 levels, although ipsilateral testicular PGE2 increased after torsion/detorsion (Figure S2).

**Figure 3:**
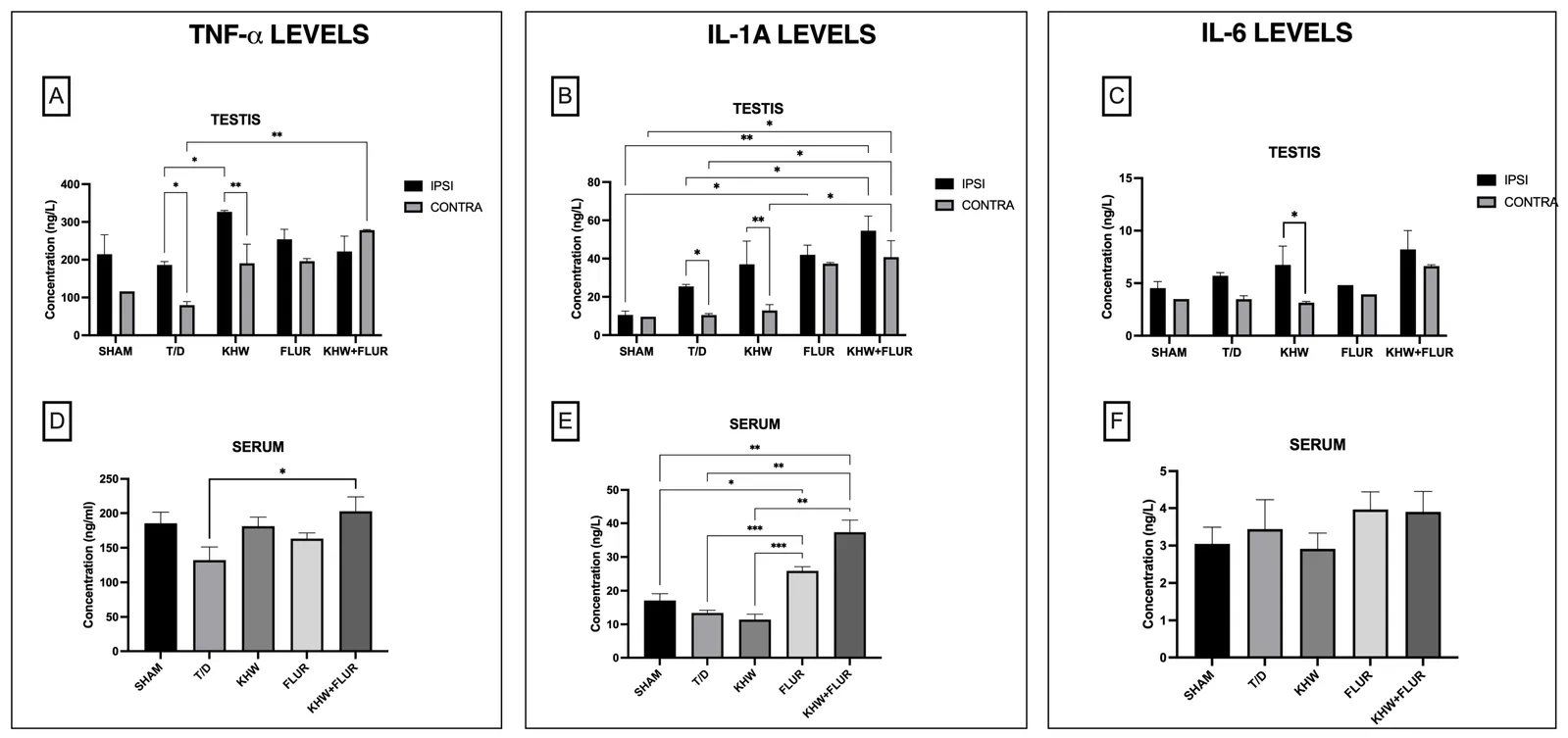
TNF-α, IL-1A, and IL-6 levels in testis and serum samples following experimental testicular torsion/detorsion. (**A**–**C**) Testicular, (**D**–**F**) serum TNF-α, IL-1A, and IL-6 levels, respectively. Data are presented as mean ± S.E.M. Black bars represent ipsilateral (IPSI) tissues, whereas gray bars represent contralateral (CONTRA) tissues. Testicular data were analyzed using mixed-effects ANOVA followed by Tukey’s post hoc test, whereas serum data were analyzed using one-way ANOVA followed by Tukey’s post hoc test. * *p* < 0.05, ** *p* < 0.01, and *** *p* < 0.001 indicate statistically significant differences (*n* = 4 per group for testicular tissues and *n* = 5–6 per group for blood samples). Statistical comparisons were performed among all experimental groups in each panel. Only statistically significant comparisons are indicated; comparisons without significance indicators were not statistically significant (*p* ≥ 0.05). (*n* = 4 per group for testicular tissues and *n* = 5–6 per group for blood samples). SHAM, sham-operated group; T/DT, torsion/detorsion group; KHW, kahweol-treated group; FLUR, flurbiprofen-treated group; KHW + FLUR, combined kahweol- and flurbiprofen-treated group.

TNF-α and IL-1α differed significantly among the experimental groups. In the ipsilateral testis, TNF-α did not increase following torsion/detorsion compared with the sham group, whereas higher levels were observed in the kahweol-treated group. Ipsilateral IL-1α showed an increasing pattern across the treatment groups, with the highest levels observed in the combination group. Serum IL-1α was increased in the flurbiprofen and combination groups, whereas IL-6 remained unchanged (Figure 3).

**Figure 4:**
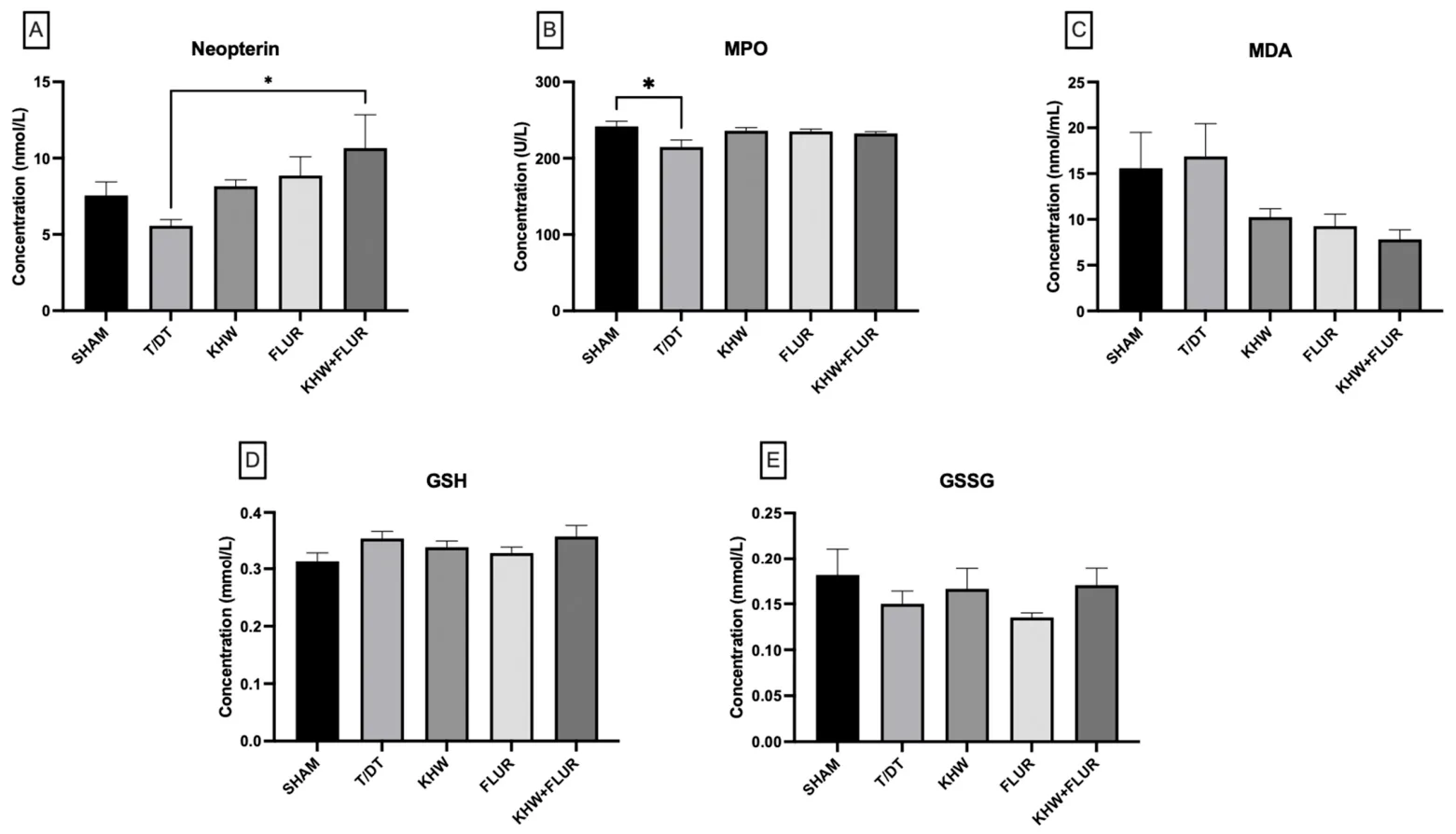
Effects of kahweol and flurbiprofen treatment on serum inflammatory and oxidative stress markers following testicular torsion/detorsion. Serum levels of (**A**) neopterin, (**B**) myeloperoxidase (MPO), (**C**) malondialdehyde (MDA), (**D**) reduced glutathione (GSH), and (**E**) oxidized glutathione (GSSG) are presented. Data are expressed as mean ± SEM. Data were analyzed using one-way ANOVA followed by Tukey’s post hoc test * *p* < 0.05 between the indicated groups. Statistical comparisons were performed among all experimental groups in each panel. Only statistically significant comparisons are indicated; comparisons without significance indicators were not statistically significant (*p* ≥ 0.05) (*n* = 5–6 per group). SHAM, sham-operated group; T/DT, torsion/detorsion group; KHW, kahweol-treated group; FLUR, flurbiprofen-treated group; KHW + FLUR, combined kahweol- and flurbiprofen-treated group.

**Figure 5:**
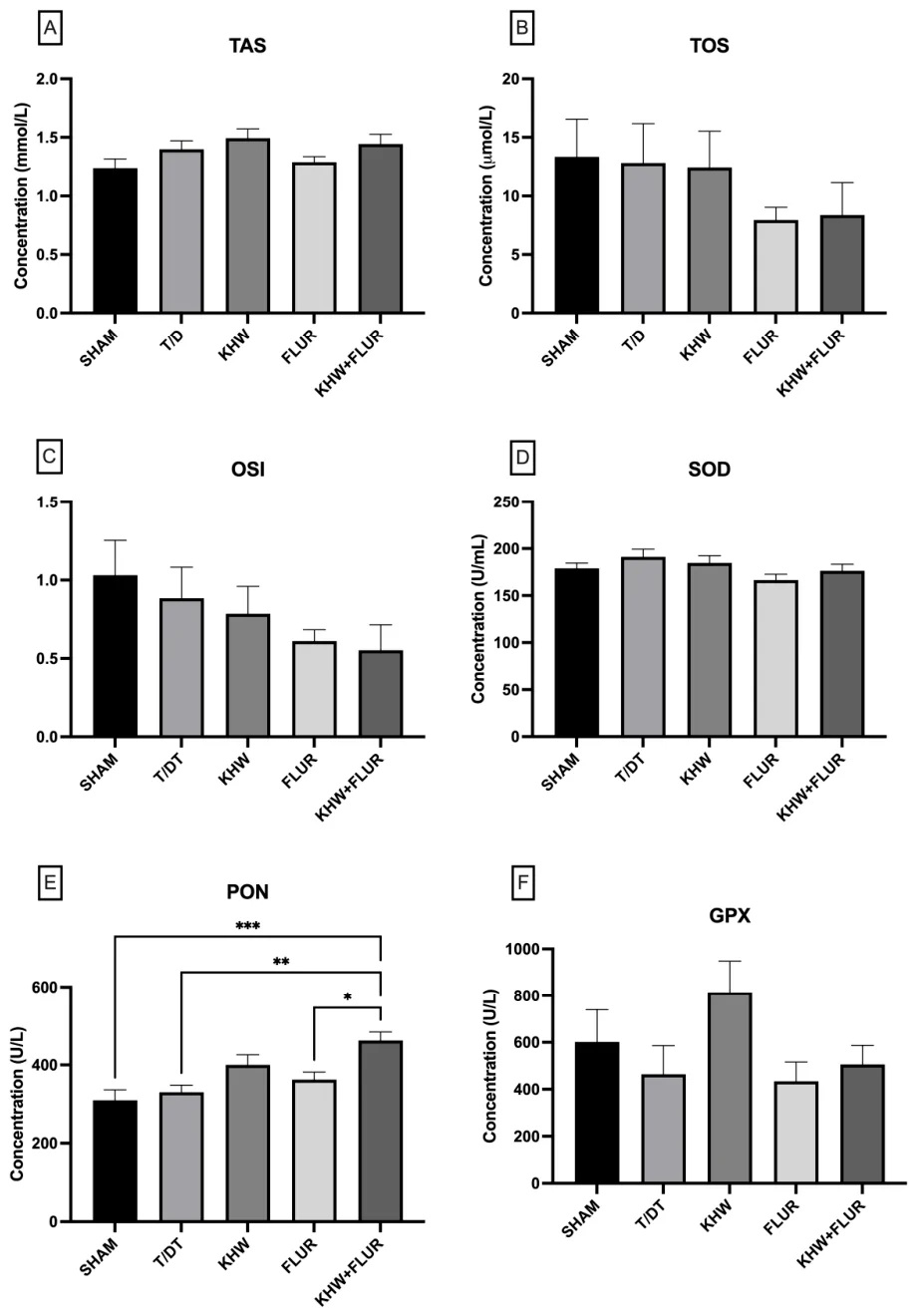
Effects of kahweol and flurbiprofen treatment on serum oxidative and antioxidative status following testicular torsion/detorsion. Serum levels of (**A**) total antioxidant status (TAS), (**B**) total oxidant status (TOS), (**C**) oxidative stress index (OSI), (**D**) superoxide dismutase (SOD), (**E**) paraoxonase (PON), and (**F**) glutathione peroxidase (GPx) are presented. Data are expressed as mean ± SEM. Data were analyzed using one-way ANOVA followed by Tukey’s post hoc test. * *p* < 0.05, ** *p* < 0.01, and *** *p* < 0.001 between the indicated groups. Statistical comparisons were performed among all experimental groups in each panel. The absence of significance indicators in panels A–D and F denotes that no statistically significant differences were detected (*p* ≥ 0.05) (*n* = 5–6 per group): SHAM, sham-operated group; T/DT, torsion/detorsion group; KHW, kahweol-treated group; FLUR, flurbiprofen-treated group; KHW + FLUR, combined kahweol- and flurbiprofen-treated group.

TAS, TOS, OSI, SOD, and GPx did not differ significantly among groups. In contrast, PON activity increased in the combination group, neopterin was elevated compared with the torsion/detorsion group, and MPO was reduced following torsion/detorsion. MDA, GSH, and GSSG remained unchanged (Figure 4 and Figure 5).

The testicular biochemical measurements were obtained from homogenate supernatants without normalization to tissue wet weight or total protein content. The statistical comparisons reported above describe differences in the measured supernatant values. However, because equivalent tissue input could not be verified, these differences cannot be interpreted as definitive quantitative changes in tissue biomarker abundance or enzyme activity. The testicular biochemical findings are therefore considered exploratory.

### 3.3. Spermatological Analyses

Spermatological analyses were limited by severe testicular and epididymal damage, particularly on the ipsilateral side, resulting in variable sample availability across groups and parameters. In the torsion/detorsion group, spermatozoa were detected in only one ipsilateral sample. Accordingly, morphology and viability could be assessed in only that sample, precluding reliable statistical comparisons of these parameters. The main findings are summarized in Table 1. These findings should be interpreted cautiously because of the limited sample availability and variability of the measurements.

### 3.4. Isolated Organ Bath Experiments

**Figure 6:**
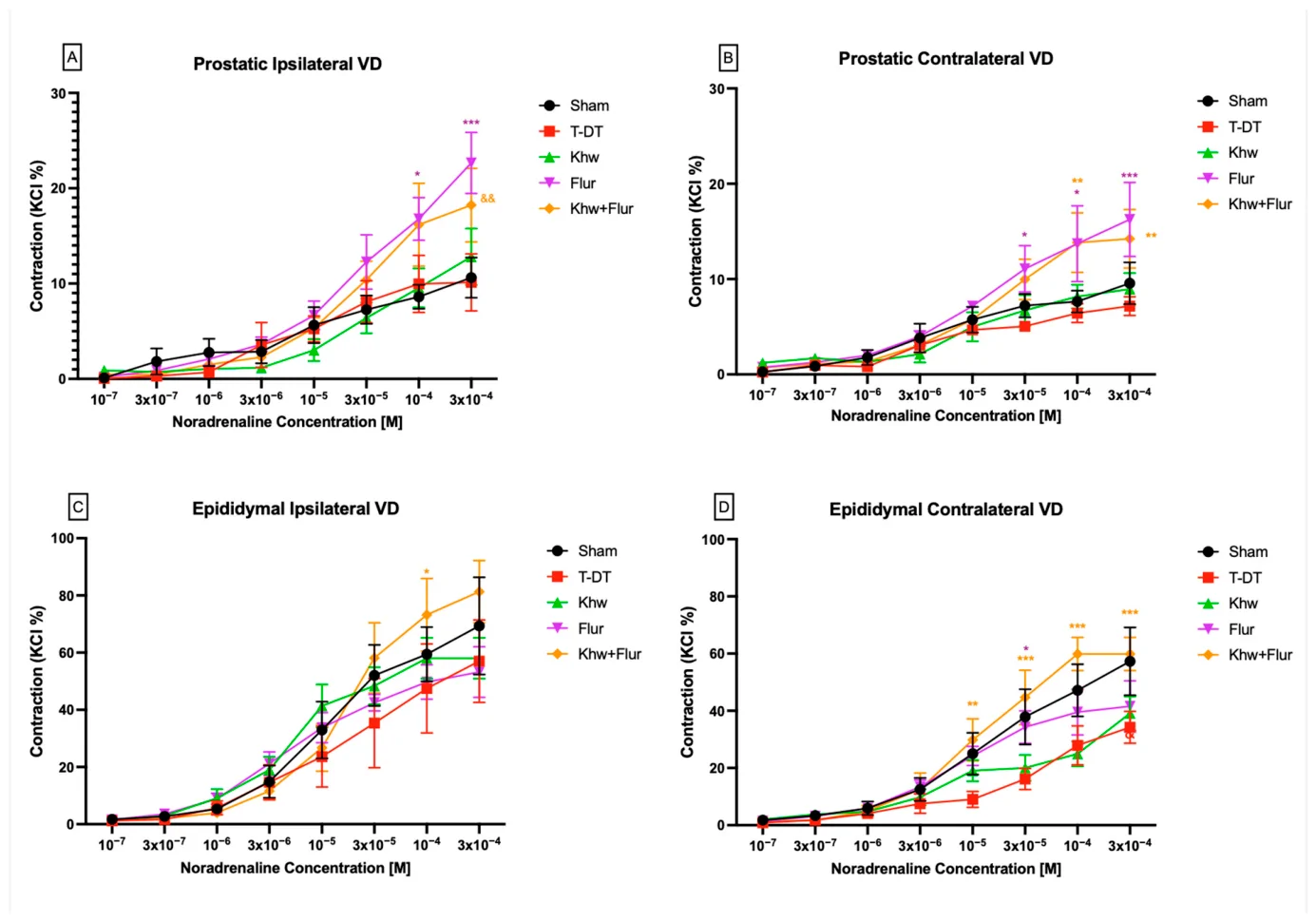
Concentration-dependent contractile responses to noradrenaline in prostatic and epididymal vas deferens tissues following experimental testicular torsion/detorsion. (**A**) Prostatic ipsilateral vas deferens. (**B**) Prostatic contralateral vas deferens. (**C**) Epididymal ipsilateral vas deferens. (**D**) Epididymal contralateral vas deferens. Contractile responses are expressed as a percentage of KCl-induced contraction (KCl %). Data are presented as mean ± S.E.M. The animal was the experimental unit. Each panel was analyzed separately using two-way repeated-measures ANOVA, with treatment group as the between-subject factor and noradrenaline concentration as the within-subject factor. Main effects and the treatment × concentration interaction were evaluated, followed by Tukey’s multiple-comparisons test. * *p* < 0.05, ** *p* < 0.01, *** *p* < 0.001: significant difference compared to the T-DT group; ^&&^ *p* < 0.01 significant difference compared to the sham group (*n* = 6 per group).

No significant differences were observed among the groups for KCl- or α,β-methylene ATP-mediated contractions. Testicular torsion/detorsion modified noradrenaline-induced contractions in both prostatic and epididymal segments of the vas deferens. In the sham group, the Emax values for noradrenaline-induced contractions were 10.63 ± 2.11% in the ipsilateral prostatic, 69.35 ± 16.98% in the ipsilateral epididymal, 9.57 ± 2.20% in the contralateral prostatic, and 57.27 ± 11.89% in the contralateral epididymal segments. Noradrenaline-induced contractile responses differed among groups in a manner that varied by tissue side, vas deferens region, and agonist concentration (Figure 6). Treatment with kahweol and/or flurbiprofen improved contractile responses in selected comparisons at specific noradrenaline concentrations (ipsilateral prostatic: kahweol %12.81 ± 2.96, flurbiprofen %22.26 ± 3.20, combined %18.23 ± 3.87; ipsilateral epididymal: kahweol %58.03 ± 7.17, flurbiprofen %53.22 ± 8.86, combined %81.30 ± 10.92; contralateral prostatic: kahweol %8.95 ± 1.68, flurbiprofen %16.26 ± 3.88, combined %14.23 ± 2.67; contralateral epididymal: kahweol %39.11 ± 5.92, flurbiprofen %41.57 ± 8.91, combined %59.89 ± 5.80) (Figure 6).

EFS-induced contractions were significantly reduced in prostatic vas deferens after torsion/detorsion, whereas combined treatment significantly enhanced responses at higher stimulation frequencies in epididymal tissues, significant differences were observed for specific group comparisons at selected stimulation frequencies, as indicated in Table 2.

## 4. Discussion

Unilateral testicular torsion is a surgical emergency caused by twisting of the spermatic cord and is typically treated by surgical detorsion. The extent of testicular damage depends on the duration and degree of torsion, and severe cases frequently result in I/R injury after detorsion due to reactive oxygen species generation, inflammatory cytokine release, and intracellular calcium overload [3,4]. In the present study, I/R injury caused marked structural damage in the ipsilateral testis, whereas the contralateral testis remained largely unaffected, supporting the successful induction of severe ipsilateral injury. Accordingly, Cosentino scores were significantly increased in all torsion groups compared with the sham group, indicating that neither kahweol, flurbiprofen, nor their combination prevented severe histopathological damage. These findings are consistent with previous studies showing that detorsion alone provides limited protection against testicular damage such as necrosis and apoptosis [25,26]. Caspase-3 expression was significantly reduced after 7 days in the kahweol, flurbiprofen, and combined treatment groups compared with the T/D group. However, in the kahweol and combined treatment groups it remained significantly higher than in the sham group, whereas in the flurbiprofen group it did not differ significantly from the sham group. The absence of a significant difference from the sham group does not establish superiority over the other treatments, and direct comparisons among the treatment groups revealed no significant differences. Therefore, the present data do not allow us to conclude that flurbiprofen exerted a stronger anti-apoptotic effect than kahweol or the combination. Reduced caspase-3 immunoreactivity should also be interpreted in the context of persistent structural damage, since extensive germ cell desquamation and necrosis may decrease the number of cells capable of expressing the marker. Nevertheless, Cosentino scores were similarly high in the T/D and treatment groups, making it less likely that the lower caspase-3 expression in the treated groups is explained solely by cell loss and suggesting a partial anti-apoptotic effect of the treatment regimens. Importantly, this reduction in the apoptotic marker was not accompanied by histological recovery; therefore, decreased caspase-3 immunoreactivity should not be equated with tissue protection. Recent studies have also highlighted the involvement of oxidative stress-sensitive signaling pathways and apoptosis in testicular I/R injury, suggesting that structural damage may persist despite pharmacological interventions [27,28].

Previous studies have shown that I/R injury during testicular torsion results from the accumulation of reactive oxygen species (ROS), which interact with cellular components such as DNA, proteins, lipids, and carbohydrates. If uncontrolled, these highly reactive molecules can cause permanent tissue damage, including DNA damage, protein denaturation, germ cell apoptosis, and lipid peroxidation [3,4,29]. Similarly, Afolabi et al. demonstrated that oxidative stress, inflammation, and apoptosis contribute to testicular torsion/detorsion injury, supporting the involvement of multiple pathways in tissue damage [7]. Untreated torsion causes testicular atrophy and degeneration, and I/R injury may still occur in approximately 25% of cases despite timely surgical intervention [30]. Testicular torsion/detorsion induces morphological and biochemical changes associated with oxidative stress and inflammation. Although the mechanisms by which antioxidants preserve male fertility are not fully understood, several studies have reported protective effects of antioxidant enzymes and pharmacological agents against testicular I/R injury [31,32,33].

Since all biochemical measurements were performed on day 7 following detorsion, the observed changes in oxidative stress and inflammatory markers reflect the subacute post-injury phase rather than the immediate acute response to ischemia–reperfusion in the present study. In the ipsilateral testis, increased COX-1 and COX-2 levels following torsion/detorsion were observed, while COX-2 remained elevated in the kahweol group. Furthermore, an ipsilateral–contralateral difference in testicular PGE_2_ was observed only in the torsion/detorsion group. However, these findings should be interpreted as exploratory biochemical observations rather than evidence of pathway-specific target engagement. Although flurbiprofen is an established COX inhibitor, COX enzymatic activity was not directly measured in the present study. Furthermore, PGE_1_, PGF_2_α, and serum PGE_2_ levels did not differ significantly among the groups. Therefore, the present biochemical findings should not be interpreted as direct evidence of COX target engagement by flurbiprofen under these experimental conditions. The cytokine findings at day 7 did not demonstrate a consistent anti-inflammatory effect of either treatment. Because inflammatory mediators were assessed only at a single subacute time point, these findings cannot characterize the acute inflammatory response following reperfusion and should therefore be interpreted cautiously.

An important limitation of the present study is the use of 10% DMSO as the vehicle. Although the same vehicle regimen was used in the sham and torsion/detorsion groups, DMSO has radical-scavenging properties and may have influenced the oxidative stress-related outcomes. Therefore, its repeated administration may have contributed to the absence of significant differences in TAS, TOS, OSI, MDA, SOD, GPx, and GSH. These negative findings should consequently be interpreted with caution and should not be considered evidence for the absence of oxidative stress following torsion/detorsion.

Endocannabinoids such as anandamide are known to be involved in various physiological processes [9]. Anandamide has been shown to exert anti-inflammatory effects through multiple mechanisms [12]. Kahweol has been reported to possess anti-inflammatory, hepatoprotective, anticancer, and antidiabetic properties and has been proposed to influence endocannabinoid signaling through FAAH-related mechanisms [13,14]. In addition to FAAH-mediated hydrolysis, anandamide can undergo COX-2-dependent metabolism to form prostaglandin ethanolamides, providing a biochemical basis for potential interactions between endocannabinoid and prostaglandin signaling [34,35]. In a traumatic brain injury model, Lee et al. demonstrated that repeated kahweol administration (5 mg/kg on day 1 followed by 1 mg/kg every 24 h) reduced leukocyte infiltration and brain injury more effectively than a single dose [15]. In the present study, no significant changes were observed in testicular FAAH or anandamide levels, while serum FAAH was increased in the kahweol group. However, FAAH was assessed by ELISA rather than by a direct enzymatic activity assay. Therefore, these findings cannot be interpreted as evidence of FAAH inhibition by kahweol. In addition, the ELISA-based measurement of anandamide represents an analytical limitation, and more specific methods such as LC–MS/MS would be required for more robust quantification. Accordingly, the present findings do not establish FAAH inhibition or increased endocannabinoid signaling by kahweol.

The unexpected decrease in MPO levels should be interpreted with caution. Since biochemical measurements were performed at a single experimental time point, the temporal course of MPO changes could not be evaluated. Consequently, this finding does not allow for definitive and comprehensive conclusions regarding the underlying inflammatory mechanisms or the dynamics of neutrophil-associated responses. Future studies involving multiple sampling time points are needed to elucidate the temporal profile and biological significance of MPO changes following testicular torsion/detorsion; accordingly, we are planning more detailed studies for the future.

Unilateral testicular torsion can impair spermatological parameters and affect both ipsilateral and contralateral vas deferens function. In addition to surgery, antioxidant and protective therapies have been investigated to reduce I/R injury and improve fertility outcomes [5,6,36]. Previous studies reported that antioxidant agents such as betaine, crocin, nitroglycerin, and cysteamine can attenuate testicular I/R injury and improve spermatological parameters when administered alongside surgical intervention [3,4,7,8]. Xia et al. 2020, demonstrated that unilateral testicular torsion may also impair contralateral testicular function and spermatogenesis, likely through I/R injury in the early phase and autoimmune mechanisms later [6]. Severe testicular and epididymal damage was observed in the torsion/detorsion group, and sperm collection was not possible in several animals, particularly from the ipsilateral side. Consequently, the spermatological findings are based on the remaining evaluable samples and should be interpreted descriptively and with caution, as they may not fully represent the respective experimental groups. Accordingly, no firm conclusions regarding treatment-related improvement in sperm parameters can be drawn from the present data.

Contraction of the vas deferens is essential for ejaculation and is regulated by multiple mediators. EFS-induced biphasic contractions are mediated primarily by ATP in the first phase and noradrenaline in the second phase, with additional modulation by neuropeptide Y, endocannabinoids, prostaglandins, and related mediators [37,38,39,40,41]. Regional differences in purinergic and adrenergic responses along the vas deferens may also contribute to the variable contractile effects observed between prostatic and epididymal segments [37]. Ventura reported that biphasic contractile responses in rodent vas deferens involve both purinergic and adrenergic neurotransmission, with additional contributions from prejunctional regulatory mechanisms [38]. Previous studies have shown that unilateral torsion/detorsion impairs vas deferens motility on both the ipsilateral and contralateral sides and that neither melatonin nor mexiletine fully restores this dysfunction [5,36]. Consistent with these observations, torsion/detorsion altered vas deferens responses to electrical field stimulation and noradrenaline, indicating persistent functional impairment at day 7 after injury without establishing specific presynaptic or postsynaptic mechanisms. In our previous in vitro study, flurbiprofen enhanced anandamide-induced EFS responses and decreased FAAH levels in the prostatic vas deferens, supporting an interaction between the endocannabinoid and cyclooxygenase systems [20]. Similarly, flurbiprofen improved noradrenaline-induced contractility in the contralateral epididymal segment after torsion/detorsion, consistent with a modulatory role of prostaglandin pathways in postsynaptic responses.

The differences between histopathological and functional outcomes may provide further insight into the effects of kahweol and flurbiprofen following testicular I/R injury. Although all treatments reduced caspase-3 immunoreactivity, severe ipsilateral histopathological damage persisted, suggesting that attenuation of apoptotic signaling alone was insufficient to prevent structural injury in this model. Interestingly, flurbiprofen and its combination with kahweol partially improved noradrenaline-induced vas deferens contractions, while combination treatment also enhanced EFS-induced responses in the prostatic segment. These findings suggest that the effects of treatment on vas deferens contractility may differ from those on testicular tissue damage. Since prostaglandins and endocannabinoids are involved in the modulation of neurotransmitter release and smooth muscle contraction, changes in these signaling pathways may contribute to the observed functional responses. However, the absence of consistent changes in FAAH, anandamide, and prostaglandin levels, together with the lack of direct measurements of COX and FAAH activities, prevents a definitive mechanistic interpretation. Therefore, the improvements observed in contractility should be considered functional effects rather than direct evidence of an interaction between cyclooxygenase and endocannabinoid pathways. Previous studies have demonstrated that endocannabinoid and cyclooxygenase pathways may interact in the regulation of vas deferens contractility [20]. Furthermore, ATP-induced prostaglandin E_2_ release has been shown to modulate electrically evoked contractions in rat vas deferens, supporting the possible involvement of prostaglandin signaling in the functional responses observed in our study [42].

The severity of the model may have caused a floor effect, limiting the detection of partial treatment effects. An important limitation of this study was the limited recovery of spermatozoa, particularly from severely damaged ipsilateral tissues. Although the same sperm collection procedure was applied to all samples, spermatozoa could not be detected in some specimens, while only limited numbers were recovered in others. In the original dataset, unevaluable sperm parameters were recorded as zero, which affected the calculated group means. Therefore, these values were treated as missing observations in the revised analyses, and the results were recalculated using evaluable samples. The limited sperm recovery, particularly in the torsion/detorsion group, may have introduced selection bias and reduced the representativeness of the findings. However, the small and unequal sample sizes and the inability to verify the exact number of spermatozoa evaluated in each sample limit the reliability of these findings. Accordingly, the spermatological results should be interpreted as exploratory, and no definitive conclusions regarding treatment-related improvements in sperm quality can be drawn. Earlier assessment time points and testicular weight measurements should be considered in future studies. Another limitation of the present study was the absence of a standardized postoperative analgesic regimen. Although all animals were monitored twice daily and no deterioration in their general condition was observed, postoperative pain and stress could not be completely excluded. Since flurbiprofen also possesses analgesic properties, differences in postoperative pain and stress between experimental groups may have contributed to the observed beneficial effects. Therefore, these effects cannot be attributed exclusively to direct tissue-protective mechanisms.

An important consideration in interpreting the testicular biochemical results is the lack of verifiable tissue-input standardization. Although all samples were processed using the same homogenization conditions and a fixed buffer volume, the individual tissue-weight records were unavailable, and the measurements were not normalized to tissue wet weight or total protein content. As a result, differences in the amount of tissue included in each homogenate may have affected the dilution of tissue-derived constituents and contributed to the observed variation in biomarker concentrations and enzyme activities. This limitation is particularly relevant to comparisons between treatment groups and between ipsilateral and contralateral testes. Yuan et al. have emphasized the importance of considering tissue heterogeneity and appropriate normalization strategies in tissue biomarker analysis. In the present study, the absence of verifiable tissue masses and an independent normalization measure prevents us from determining whether the observed differences in testicular homogenate values reflect biological variation, differences in tissue input, or a combination of both [43]. We have therefore reconsidered the interpretation of the testicular biochemical findings. These measurements are retained as exploratory observations, but they cannot independently establish treatment-related changes in tissue oxidative stress, inflammatory mediator abundance, or enzyme activity. Likewise, they do not provide sufficient evidence to attribute specific antioxidant, anti-inflammatory, or molecular effects to kahweol, flurbiprofen, or their combination. This distinction is important because statistical differences in non-normalized homogenate values do not necessarily establish equivalent differences per unit of tissue. The limitation could not be resolved retrospectively because the original tissue-weight records were unavailable and no residual homogenate supernatants remained for additional protein measurements. The revised interpretation follows the broader principle of transparent methodological reporting and appropriately qualified conclusions emphasized in the ARRIVE 2.0 guidelines [44]. Future studies should prospectively record the tissue mass used for each homogenate and apply an analytically appropriate normalization strategy. The serum biochemical findings are considered separately because the tissue-input normalization issue does not apply to serum samples.

In conclusion, testicular torsion/detorsion caused severe ipsilateral testicular damage and persistent impairment of vas deferens contractility at day 7. Although kahweol, flurbiprofen, and their combination reduced caspase-3 immunoreactivity, none of the treatments significantly improved the overall histopathological damage. Flurbiprofen and combination treatment partially improved noradrenaline-induced contractions, while the combination enhanced EFS-induced responses in the prostatic vas deferens. However, the biochemical findings did not provide sufficient evidence to confirm the involvement of specific cyclooxygenase or endocannabinoid mechanisms. These findings suggest that flurbiprofen, alone or combined with kahweol, may partially improve selected functional outcomes following testicular ischemia–reperfusion injury, despite persistent structural damage. The testicular biochemical findings remain exploratory because equivalent tissue input could not be verified and the homogenate measurements were not normalized to tissue wet weight or total protein content. Consequently, these results cannot independently substantiate treatment-related antioxidant or anti-inflammatory effects or specific molecular mechanisms.

## Figures and Tables

**Table 1:** Summary of spermatological parameters following experimental testicular torsion/detorsion. Data are presented as mean ± SEM where applicable, results based on a single animal are reported as individual values without SEM. Given the variable availability of sperm samples, the results should be interpreted with caution. No inferential statistical analyses were performed on the spermatological data because of the limited and unequal sample sizes.

Parameter	Sham		Torsion/Detorsion		Kahweol		Flurbiprofen		Kahweol + Flurbiprofen	
	Ipsilateral *n* = 6	Contralateral *n* = 6	Ipsilateral *n* = 1	Contralateral *n* = 3	Ipsilateral	Contralateral *n* = 6	Ipsilateral	Contralateral *n* = 6	Ipsilateral	Contralateral *n* = 6
Concentration (million/mL)	2.05 ± 0.54	4.39 ± 0.51	1.57	5.95 ± 1.31	1.08 ± 0.37 (*n* = 6)	5.84 ± 1.02	4.47 ± 1.10 (*n* = 6)	7.01 ± 0.76	1.51 ± 0.78 (*n* = 6)	6.77 ± 0.66
Total motility (%)	29.4 ± 8.6	50.54 ± 4.64	37.50	50.86 ± 10.16	31.48 (*n* = 1)	59.26 ± 4.85	49.76 ± 5.32 (*n* = 2)	55.55 ± 5.96	26.74 (*n* = 1)	62.80 ± 2.97
Progressive motility (%)	28.02 ± 6.37	26.13 ± 3.37	20.83	28.21 ± 8.36	11.11 (*n* = 1)	34.45 ± 4.09	18.82 ± 10.18 (*n* = 2)	25.19 ± 4.76	4.65 (*n* = 1)	32.76 ± 2.93
Normal morphology (%)	44.04 ± 9.37	64.11 ± 3.47	44.44	72.25 ± 2.22	35.71 (*n* = 1)	67.24 ± 3.45	41.08 ± 10.76 (*n* = 6)	69.98 ± 2.47	20.46 ± 6.46 (*n* = 2)	71.9 ± 1.96

**Table 2:** Electrical field stimulation (EFS)-induced contractile responses in ipsilateral and contralateral prostatic and epididymal vas deferens tissues following experimental testicular torsion/detorsion. Responses are presented as peak-to-peak (PP) contraction amplitude (g) and area under the contraction curve (g·s). Data are presented as mean ± SEM. Each animal constituted one biological replicate. Responses were recorded at stimulation frequencies of 2, 4, 8, 16, and 32 Hz. Each tissue side and region was analyzed separately using two-way repeated-measures ANOVA, with treatment group as the between-subject factor and stimulation frequency as the within-subject factor. Main effects and the treatment × frequency interaction were evaluated, followed by Tukey’s multiple-comparisons test. * *p* < 0.05, ** *p* < 0.01 versus SHAM; ^&^ *p* < 0.05 versus T/DT; ^#^ *p* < 0.05, ^##^ *p* < 0.01 versus KHW.

VD	Group	2 Hz		4 Hz		8 Hz		16 Hz		32 Hz	
		P-P	Area	P-P	Area	P-P	Area	P-P	Area	P-P	Area
Prostatic ipsilateral	Sham	15.28 ± 5.45	104.53 ± 50.5	18.86 ± 5.57	139.15 ± 62.5	27.74 ± 9.89	191.21 ± 7.58	45.49 ± 13.39	338.68 ± 108.03	76.61 ± 17.41	592.47 ± 121.11
	T/DT	4.6 ± 1.89	16.81 ± 7.59	3.69 ± 1.08	23.84 ± 10.53	5.31 ± 1.64	35.12 ± 14.65	14.65 ± 3.44 *	103.41 ± 32.82 *	48.9 ± 13.11 *	391.7 ± 110.69
	Khw	5.41 ± 1.32	8.56 ± 2.33	4.24 ± 0.88	10.7 ± 3.56	5.01 ± 0.9	17.95 ± 4.18	8.06 ± 1.51	68.01 ± 21.58	40.87 ± 8.53	361.47 ± 73.35
	Flur	1.73 ± 0.43	10.66 ± 4.11	3.14 ± 0.75	18.53 ± 4.53	7.7 ± 1.89	64.28 ± 18.19	21.5 ± 4.55	181.31 ± 43.97	56.69 ± 6.18	524.04 ± 76.23
	Khw + Flur	3.24 ± 0.75	14.52 ± 5.24	4.84 ± 1.77	34.57 ± 17.29	12.84 ± 6.55	133.42 ± 4.03	31.63 ± 15.51	329.69 ± 210.4	76.8 ± 23.72 ^&,##^	767.96 ± 349.62 ^&,#^
Epididymal ipsilateral	Sham	23.71 ± 10.74	165.08 ± 6.12	39.87 ± 17.59	353.99 ± 160.97	89.62 ± 41.85	735.55 ± 332.31	165.56 ± 8.30	1713.02 ± 910.60	225.74 ± 73.47	2252.49 ± 775.82
	T/DT	7.31 ± 2.06	51.48 ± 14.32	8.55 ± 2.17	77.23 ± 22.63	23.75 ± 9.59	207.49 ± 92.16	85.53 ± 7.85	621.08 ± 219.65	197.24 ± 49.26	1831.83 ± 493.21
	Khw	5.27 ± 1.40	23.87 ± 9.51	6.82 ± 1.58	43.07 ± 14.65	20.09 ± 6.85	112.90 ± 45.40	80.88 ± 3.66	591.49 ± 190.45	180.82 ± 31.87	1682.62 ± 329.76
	Flur	6.54 ± 1.99	30.21 ± 5.99	12.60 ± 3.17	58.98 ± 11.90	35.92 ± 10.28	332.21 ± 67.64	126.23 ± 35.62	998.34 ± 257.50	247.72 ± 53.63	2477.86 ± 433.64
	Khw + Flur	5.95 ± 1.23	32.68 ± 12.36	11.17 ± 3.70	79.41 ± 35.22	33.34 ± 8.64	215.48 ± 61.44	132.14 ± 5.16	2106.10 ± 1086.22 ^&,#^	237.61 ± 7.85	2706.57 ± 803.43
Prostatic contralateral	Sham	19.09 ± 5.93	119.86 ± 1.50	21.13 ± 6.34	161.69 ± 60.11	33.12 ± 11.57	241.46 ± 79.08	56.66 ± 16.65	420.75 ± 145.21	84.96 ± 18.09	658.95 ± 151.73
	T/DT	8.60 ± 3.66	40.26 ± 16.54	7.26 ± 2.17	63.74 ± 35.14	5.96 ± 1.22 *	53.42 ± 18.14	19.47 ± 5.39 **	127.52 ± 34.87 *	45.18 ± 5.16 **	338.02 ± 80.90
	Khw	8.45 ± 2.82	23.12 ± 7.84	7.86 ± 3.52	24.60 ± 8.50	9.58 ± 4.10	28.76 ± 9.45	13.46 ± 3.39	65.12 ± 24.92	46.27 ± 7.93	323.86 ± 58.16
	Flur	2.82 ± 0.92	6.99 ± 2.65	4.59 ± 1.26	16.83 ± 6.39	11.32 ± 4.27	77.30 ± 36.60	24.02 ± 9.15	211.25 ± 86.50	50.65 ± 13.00	476.49 ± 149.73
	Khw + Flur	7.12 ± 2.74	18.86 ± 5.55	9.27 ± 3.10	30.48 ± 11.80	16.58 ± 5.06	100.79 ± 52.28	31.68 ± 14.84	301.06 ± 184.81	73.62 ± 24.78 ^&^	728.23 ± 343.25 ^&,#^
Epididymal contralateral	Sham	34.60 ± 13.88	219.34 ± 3.94	60.80 ± 21.70	451.87 ± 71.65	164.88 ± 7.09	1254.15 ± 430.21	306.19 ± 0.22	2878.15 ± 706.30	371.72 ± 72.10	3837.16 ± 771.01
	T/DT	12.47 ± 4.65	148.71 ± 2.57	16.32 ± 8.27	160.65 ± 07.82	72.46 ± 52.35	539.89 ± 398.30	202.08 ± 110.20	1585.99 ± 928.04	339.58 ± 122.50	3366.09 ± 1398.93
	Khw	11.54 ± 2.85	96.30 ± 53.11	10.24 ± 2.20	73.93 ± 24.28	35.68 ± 13.32	168.78 ± 61.85	178.29 ± 56.74	1147.45 ± 389.61	367.20 ± 73.27	3291.72 ± 667.99
	Flur	10.32 ± 2.08	51.44 ± 13.37	21.07 ± 5.25	94.36 ± 34.49	55.00 ± 24.82	382.27 ± 168.79	162.11 ± 44.89	1360.16 ± 477.40	298.39 ± 50.86	2902.15 ± 547.48
	Khw + Flur	11.26 ± 4.95	63.08 ± 43.10	22.21 ± 9.62	132.68 ± 78.16	77.28 ± 30.25	474.01 ± 192.12	202.33 ± 68.99	1693.02 ± 634.11	326.53 ± 64.29	3499.39 ± 745.32

## Data Availability

Data will be made available upon reasonable request.

## Supplementary Materials

The following supporting information can be downloaded at: https://www.mdpi.com/article/10.3390/molecules31213922/s1, Table S1. Animal allocation, sample availability, and effective sample sizes for each analysis. All n values represent individual animals, not technical replicates. For biochemical analyses, the reported range indicates the minimum and maximum numbers of animals contributing to individual analyses across groups, sample types, tissue sides, and parameters; it does not indicate a uniform sample size for every comparison. The sample size calculation indicated a minimum of six animals per group. Eight animals were allocated to each analytical subgroup to accommodate an anticipated mortality rate of approximately 20%. For isolated organ bath analyses in which six animals per group were evaluated, no additional samples were analyzed after this target was reached. Samples not analyzed for this reason were distinct from those unavailable or unevaluable because of technical problems or tissue injury. Figure S1. Fatty acid amide hydrolase (FAAH) and anandamide levels in testis and serum. Data are presented as mean ± S.E.M. Black bars represent ipsilateral (IPSI) tissues, whereas gray bars represent contralateral (CONTRA) tissues. Testicular data were analyzed using mixed-effects ANOVA followed by Tukey’s post hoc test, whereas serum data were analyzed using one-way ANOVA followed by Tukey’s post hoc test. ** *p* < 0.01, and *** *p* < 0.001 indicate statistically significant differences. Figure S2. PGE1, PGE2, and PGF2α levels in testis and serum samples following experimental testicular torsion/detorsion. (A–C) Testicular, (D–F) Serum PGE1, PGE2, and PGF2α levels, respectively. Data are presented as mean ± S.E.M. Black bars represent ipsilateral (IPSI) tissues, whereas gray bars represent contralateral (CONTRA) tissues. Testicular data were analyzed using mixed-effects ANOVA followed by Tukey’s post hoc test, whereas serum data were analyzed using one-way ANOVA followed by Tukey’s post hoc test. ** *p* < 0.01 indicates statistically significant differences.
