# Supplementary material for: Investigation of the Effects of Kahweol, Flurbiprofen and Co-Administration of Kahweol and Flurbiprofen on Vas Deferens, Testis and Sperm Against Ischemia-Reperfusion Injury Induced by Testicular Torsion and Detorsion in Rats
Source: Molecules. 2026 Oct 31;31(21):3922. doi: 10.3390/molecules31213922 (PMC13765252; doi:10.3390/molecules31213922)
Supplement: Supplementary file 1 [file molecules-31-03922-s001.zip › molecules-4615579-supplementary.pdf]

molecules

Article

Investigation of the Effects of Kahweol, Flurbiprofen and Co-Administration of Kahweol and Flurbiprofen on Vas Deferens, Testis and Sperm Against Ischemia-Reperfusion Injury Induced by Testicular Torsion and Detorsion in Rats

Okan Ali Aksoy [1,*], Serdar Moralioğlu [2], Yagmur Okcay [3], Berk Alp Goksel [1], Süheyla Esra Ozkocer [4], Kemal Tuna Olgac [5], Bekir Erdeve [6], Elif Bersu Gul [5], Berkay Bagci [4], Sema Kocabiyik [7], Elif Hilal Vural [8], Gokce Sevim Ozturk Fincan [9], Emre Avci [7], Onur Erdem [10] and Ismail Mert Vural [3]

1 Department of Animal Experiments and Research Centre, Gulhane Institute of Health Sciences, University of Health Sciences, Ankara 06010, Türkiye; berkalp.goksel@sbu.edu.tr
2 Department of Pediatric Surgery, İstanbul Zeynep Kamil Maternity and Children Diseases Health Training and Research Center, University of Health Sciences, İstanbul 34668, Türkiye; serdar.moralioglu@sbu.edu.tr
3 Department of Pharmacology, Gulhane Faculty of Pharmacy, University of Health Sciences, Ankara 06010, Türkiye; yagmur.okcay@sbu.edu.tr (Y.O.); ismailmert.vural@sbu.edu.tr (I.M.V.)
4 Department of Medical Histology, Faculty of Medicine, Gazi University, Ankara 06500, Türkiye; sesavari@gmail.com (S.E.O.); berkay421998@gmail.com (B.B.)
5 Department of Reproduction and Artificial Insemination, Faculty of Veterinary Medicine, Ankara University, Ankara 06070, Türkiye; ktolgac@gmail.com (K.T.O.); ebersugul@gmail.com (E.B.G.)
6 Department of Pediatric Surgery, Faculty of Medicine, Hacettepe University, Ankara 06100, Türkiye; bekirerdeve@gmail.com
7 Department of Biochemistry, Gulhane Faculty of Pharmacy, University of Health Sciences, Ankara 06010, Türkiye; semanurkayali@hotmail.com (S.K.); emre.avci@sbu.edu.tr (E.A.)
8 Department of Medical Pharmacology, Faculty of Medicine, Lokman Hekim University, Ankara 06510, Türkiye; elif.vural@lokmanhekim.edu.tr
9 Department of Medical Pharmacology, Faculty of Medicine, Gazi University, Ankara 06500, Türkiye; gsozturk@gazi.edu.tr
10 Deparment of Pharmaceutical Toxicology, Gulhane Faculty of Pharmacy, University of Health Sciences, Ankara 06010, Türkiye; onur.erdem@sbu.edu.tr
* Correspondence: okanali.aksoy@sbu.edu.tr

Supplementary Materials

Experimental group	Subgroup	Sample part (For each group)	Relevant Figure or Table	Animals included in analysis (n)	Reason for missing samples, missing measurements, or exclusion
1. SHAM (n=16)<br>2. T/DT (n=16)<br>3. KHW (n=16)<br>4. FLUR (n=16)<br>5. KHW + FLUR (n=16)<br>Total = 80	Histological analyses	Testis, both sides, n=8	Fig. 1	SHAM: 6; T/DT: 6; KHW: 6; FLUR: 6; KHW+FLUR: 7.	Fixation or tissue-processing problems; tissue loss, tearing, or folding during sectioning.
	Isolated organ bath experiments	Vas deferens, both sides, n=8,<br>Each cut in half to prostatic and epididymal portions	Fig.6, Table 2	n = 6 animals per group for each tissue side and region.	Absence of contractile response in viability assessment (80 mM KCl).
	Biochemical analyses, ELISA measurements	Serum, n=16<br>Testis, both sides, n=8	Fig.2, Fig.3, Fig.4, Fig.5	n = 4 per group for testicular tissues and n = 5-6 per group for blood samples	Insufficient sample volume for all assays after tissue homogenates and plasma were divided among multiple biochemical measurements.
	Spermatological analyses	Epididymis, both sides, n=8	Table 1, Supplementart Table 2.	Exact evaluable sample sizes are reported for each group, side, and parameter in Table 1. No inferential statistical comparisons were performed.	Failure to recover spermatozoa; insufficient sperm numbers for morphology or viability assessment.

Table S1. Animal allocation, sample availability, and effective sample sizes for each analysis.

All n values represent individual animals, not technical replicates. For biochemical analyses, the reported range indicates the minimum and maximum numbers of animals contributing to individual analyses across groups, sample types, tissue sides, and parameters; it does not indicate a uniform sample size for every comparison. The sample size calculation indicated a minimum of six animals per group. Eight animals were allocated to each analytical subgroup to accommodate an anticipated mortality rate of approximately 20%. For isolated organ bath analyses in which six animals per group were evaluated, no additional samples were analyzed after this target was reached. Samples not analyzed for this reason were distinct from those unavailable or unevaluable because of technical problems or tissue injury.

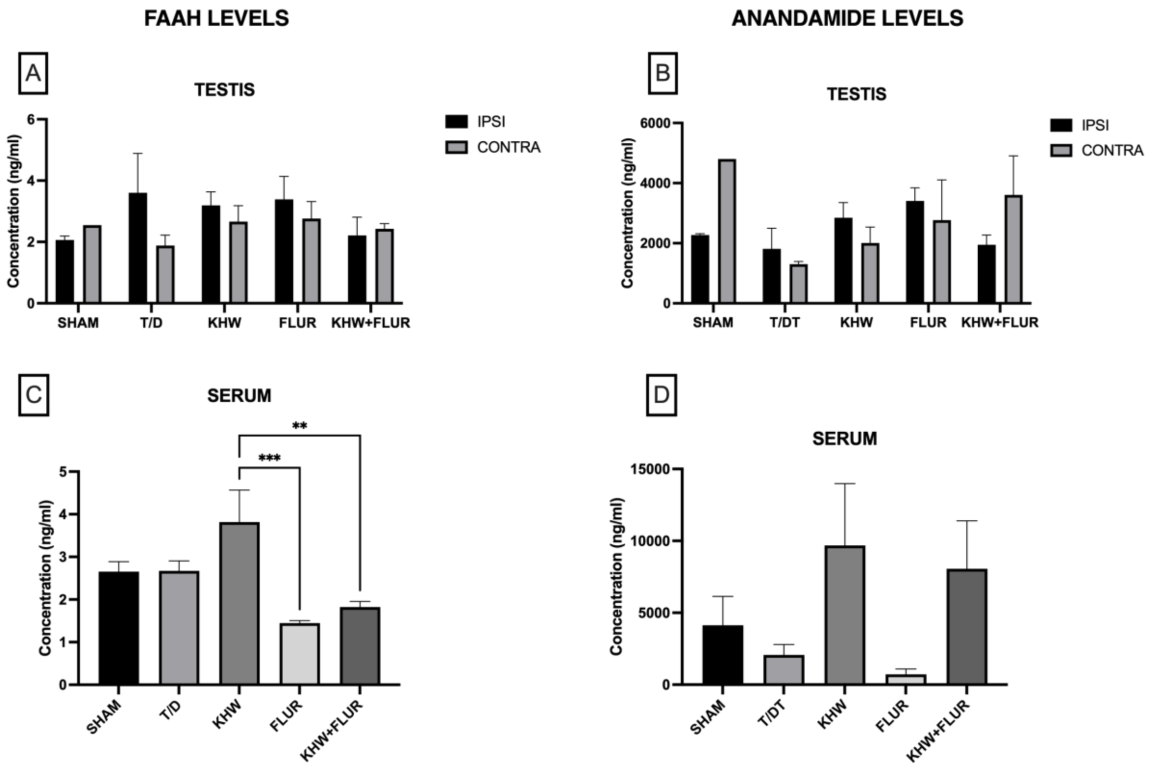

Figure S1. Fatty acid amide hydrolase (FAAH) and anandamide levels in testis and serum. Data are presented as mean ± S.E.M. Black bars represent ipsilateral (IPSI) tissues, whereas gray bars represent contralateral (CONTRA) tissues. Testicular data were analyzed using mixed-effects ANOVA followed by Tukey's post-hoc test, whereas serum data were analyzed using one-way ANOVA followed by Tukey's post-hoc test. $^{**}p<0.01$, and $^{***}p<0.001$ indicate statistically significant differences. Statistical comparisons were performed among all experimental groups in each panel. Only statistically significant comparisons are indicated; comparisons without significance indicators were not statistically significant ($p \geq 0.05$) (n = 4 per group for testicular tissues and n = 5-6 per group for blood samples). SHAM, sham-operated group; T/DT, torsion/detorsion group; KHW, kahweol-treated group; FLUR, flurbiprofen-treated group; KHW+FLUR, combined kahweol- and flurbiprofen-treated group.

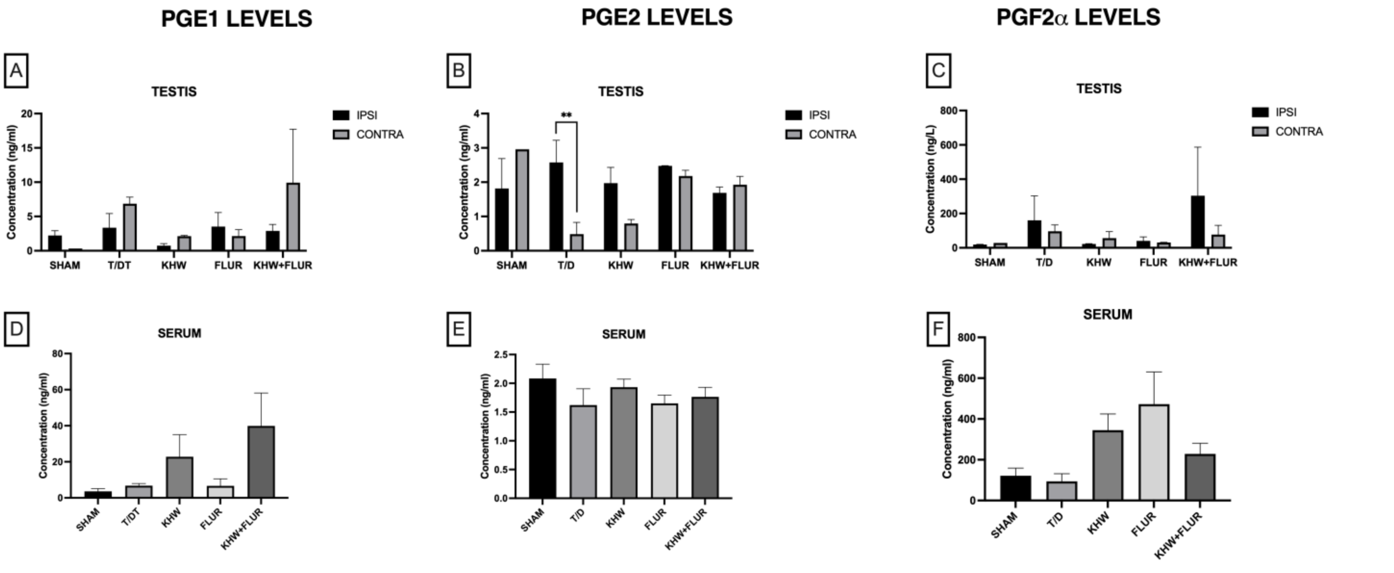

Figure S2. PGE1, PGE2, and PGF2α levels in testis and serum samples following experimental testicular torsion/detorsion. **(A–C)** Testicular, **(D–F)** Serum PGE1, PGE2, and PGF2α levels, respectively. Data are presented as mean ± S.E.M. Black bars represent ipsilateral (IPSI) tissues, whereas gray bars represent contralateral (CONTRA) tissues. Testicular data were analyzed using mixed-effects ANOVA followed by Tukey's post-hoc test, whereas serum data were analyzed using one-way ANOVA followed by Tukey's

post-hoc test. **$p<0.01$ indicates statistically significant differences. Statistical comparisons were performed among all experimental groups in each panel. Only statistically significant comparisons are indicated; comparisons without significance indicators were not statistically significant ($p \geq 0.05$) ($n = 4$ per group for testicular tissues and $n = 5$-6 per group for blood samples). SHAM, sham-operated group; T/DT, torsion/detorsion group; KHW, kahweol-treated group; FLUR, flurbiprofen-treated group; KHW+FLUR, combined kahweol- and flurbiprofen-treated group.
